# Identification and evaluation of a core microsatellite panel for use in white-tailed deer (*Odocoileus virginianus*)

**DOI:** 10.1186/s12863-019-0750-z

**Published:** 2019-06-06

**Authors:** William L. Miller, Jessie Edson, Peter Pietrandrea, Cassandra Miller-Butterworth, W. David Walter

**Affiliations:** 1 Pennsylvania Cooperative Fish and Wildlife Research Unit, Department of Ecosystem Science and Management, Intercollege Graduate Degree Program in Ecology, The Pennsylvania State University, University Park, PA USA; 2 Pennsylvania Cooperative Fish and Wildlife Research Unit, The Pennsylvania State University, University Park, PA USA; 3 Penn State Beaver, Monaca, PA USA; 4 U.S. Geological Survey, Pennsylvania Cooperative Fish and Wildlife Research Unit, The Pennsylvania State University, University Park, PA USA; 5 Present Address: Calvin College Department of Biology, 1726 Knollcrest Circle SE, Grand Rapids, MI 49546 USA

**Keywords:** White-tailed deer, *Odocoileus virginianus*, Short-tandem repeats, Microsatellites, Panel evaluation

## Abstract

**Background:**

Microsatellite loci have been used extensively over the past two decades to study the genetic characteristics of non-model species. The ease of microsatellite development and ability to adapt markers from related species has led to the proliferation of available markers for many commonly studied species. Because it is often infeasible to genotype individuals across all available loci, researchers generally rely on subsets of markers. Marker choice can bias inferences made using disparate suites of loci. This has been a primary motivation for efforts to identify uniform marker panels. Here, we use the geographic distribution of previous studies to identify microsatellite loci for white-tailed deer (*Odocoileus virginianus*) with the potential for widespread use, and we evaluate the effectiveness of this panel in a portion of the range where few previous studies have been conducted. The purpose was to consolidate the numerous genetic resources for this species into a manageable panel and to provide a uniform methodology that improves comparisons between past and future studies.

**Results:**

We reviewed microsatellite panels from 58 previous or ongoing projects and identified 106 candidate loci. We developed a multiplex protocol and evaluated the efficacy of 17 of the most commonly used loci using 720 DNA samples collected from the Mid-Atlantic region of the United States of America. Amplification errors were detected in six of these loci. The 11 remaining loci were highly polymorphic, exhibited low frequencies of null alleles, and were easy to interpret with the aid of allele binning software.

**Conclusions:**

The development of broadly-applicable, core microsatellite panels has the potential to improve repeatability and comparative ability for commonly studied species. The properties of the consolidated 11 microsatellite panel suggest that they are applicable for many common research objectives for white-tailed deer. The geographic distribution of previous studies using these markers provides a greater degree of confidence regarding the robustness to common sources of error related to amplification anomalies, such as null alleles, relative to loci with more limited use. While this does not replace further evaluation of genotyping errors, it does provide a common platform that benefits future research studies.

**Electronic supplementary material:**

The online version of this article (10.1186/s12863-019-0750-z) contains supplementary material, which is available to authorized users.

## Background

### Microsatellite markers

Genetic aspects of population dynamics and health have long been considered by biologists. Until recently, there has been a general lack of genetic and genomic tools to study species outside of a few model organisms (e.g. *Drosophila melanogaster*, *Mus musculus*, etc.). The past two decades have seen a proliferation of genetic markers that can be used to study the genetic characteristics of species with little or no a priori knowledge regarding the sequence of an organism’s genome. Genetic techniques continue to improve and have been used for a variety of applications, including population genetic analysis [1, 2], assessment of mating systems, parentage, and relatedness [3, 4], the indirect estimation of demographic parameters [5, 6], and the assessment of population viability [7–9].

Perhaps no class of genetic markers has seen more use in the last 15 years than microsatellite loci. Microsatellite markers are short, repetitive DNA sequence elements that are highly polymorphic and exhibit heterozygosity [10, 11]. These markers are common genetic elements and are widely distributed across the genome of most eukaryotes, making them an effective tool for estimating patterns of genetic diversity at a genome-scale [12]. Microsatellites have greater power per locus due to their high rate of polymorphism, when compared to biallelic markers such as single nucleotide polymorphisms (SNPs), and codominant status, when compared to dominant markers such as amplified length fragment polymorphisms (AFLPs) [10, 13, 14]. From a practical standpoint, the widespread adoption of microsatellite markers has been facilitated, in part, by the lower cost and relative ease of implementation compared to other methods. Discovery of novel microsatellite loci can be done by screening limited genomic libraries for common repeat motifs [15], although next-generation sequencing technologies can expedite the process of microsatellite development [16]. Previously developed loci can also be used in studies of closely related taxa or populations because microsatellites and flanking regions are highly conserved among these groups [17]. This allows for the use of these previously discovered microsatellites in future studies, thereby circumventing the need for the discovery of additional loci. These factors make microsatellites a cost-effective tool for studying the demography, genetic dynamics, and health of non-model species.

Despite their widespread use in contemporary genetic studies, there are several considerations that must be made when creating an effective microsatellite panel. Appropriate marker selection is imperative to obtain accurate and reproducible estimates of population structure, genetic diversity, or individual assignment. Many species, and in particular, those species that are commonly studied and/or have wide geographic ranges, often have multiple suites of candidate loci available (for example, Atlantic salmon [18–22]). Excessive genotyping efforts are costly and provide diminishing returns for common genetic analyses [14, 23, 24], which often leads to research groups subsampling a more limited number of loci. Marker choice is known to affect estimates of genetic diversity, so it is possible that more limited but disparate suites of loci may produce substantially different results, limiting the reproducibility of individual studies and even leading to erroneous conclusions in comparative studies where marker characteristics vary substantially [25]. Efforts to identify uniform microsatellite panels for commonly studied species have been identified as being of particular importance in limiting such sources of error and bias [22, 26], although they are rarely undertaken.

Several methodological factors have been cited as limiting the feasibility of efforts to identify uniform microsatellite panels. First, summarizing genetic patterns using microsatellite loci across a species’ geographic range would require significant genotyping effort, making these efforts costly and infeasible for many species that are common and widely distributed [26]. Further, it is often outside of the objectives of many genetic studies focused on population-scale questions to evaluate the efficacy of the chosen microsatellite panel across the geographic range of the species of interest. Genotyping errors caused by mutations, such as null alleles (alleles that fail to amplify due to mutations in the primer-binding regions [27]), are known to arise in some spatially-distinct localities and populations but not others [26]. Therefore, panels developed and evaluated for regional objectives may not necessarily be effective in new areas, leading to the need for additional loci. Second, certain loci may accrue insertion/deletion mutations, which alter the expected repeat motif (imperfect repeats [28]). These errors can lead to deviations from true population allele frequencies if not addressed [29]. Two final concerns relate to the relative subjectivity of the allele scoring process, which can be affected by user interpretation of electropherograms [30, 31] and differences among genotyping platforms [32, 33].

The appropriate selection of microsatellite markers paired with a careful evaluation of their efficacy can minimize potential sources of error and bias and ensure that inferred genetic patterns are comparable among studies. Additionally, proper documentation of scoring practices, polymerase chain reaction (PCR) conditions, and genotyping error rates can minimize errors associated with user interpretation. Efforts to identify a uniform suite of microsatellite loci are of most benefit if they are carried out before the implementation of regional studies. This is rarely done, and there are few guiding principles in place to facilitate the creation of a microsatellite panel that is broadly applicable, in both utility and geographic scope, from previously available but disparate suites of loci.

### Objectives

Here, we summarize the extensive number of microsatellite loci previously developed or adapted from other species for white-tailed deer (*Odocoileus virginianus*). Our goals were to identify a core microsatellite panel that can be utilized across the range of the species and for a variety of applications (e.g. population genetics, individual assignment, parentage analysis, etc.) and to detail a uniform methodology in order to improve repeatability and comparative efforts. White-tailed deer are an extensively managed and studied species with a wide geographic distribution spanning North and South America [34]. Because they are so extensively studied and managed, numerous microsatellite loci have been applied to genetic studies of white-tailed deer. While previous studies have attempted to identify a manageable panel of loci, evaluations of the proposed panels were typically limited to individual populations or geographic regions (southern Oklahoma [35], Mississippi [36], and Nebraska [37]). These regions constitute a small portion of the range of white-tailed deer, so while these studies may provide guidance for future studies in proximity to their study region, they may not be as effective in other areas across the range of white-tailed deer. While we also evaluate the effectiveness of our proposed panel in a subset of this species’ range (the Mid-Atlantic region of the United States of America), we make use of the geographic distribution of previous studies to select markers that have the potential for broad geographic applicability. Markers were selected with respect to the proportion of the species’ range where they were previously demonstrated to be effective and exhibit low rates of genotyping errors. Markers demonstrated to be effective over broad geographic distributions were predicted to have lower potential of population-scale idiosyncrasies due to the conservation of microsatellite and flanking sequences among related groups and were therefore considered favorable when compared to markers with more confined distributions. We collected samples from a region with relatively few genetic studies to test this prediction. The proposed panel was evaluated based on efficacy and interpretability.

## Methods

### Selection of microsatellite loci

We performed a literature search for articles published between 2000 and 2017 that utilize microsatellite markers to assess white-tailed deer genetics using Google Scholar (https://scholar.google.com). A combination of key words from the following categories were used as search phrases to find academic papers: species’ name (key words: ‘white-tailed deer’ or ‘*Odocoileus virginianus*’), marker type (key words: ‘microsatellites’ or ‘short-tandem repeats’), and/or application (key words: ‘population genetics’ or ‘landscape genetics’). We recorded the microsatellite loci used in each study. Studies were then grouped by geographic region and application. Geographic information was collected for each study to assess the distribution and regional effectiveness of existing loci panels. We also summarized the application for which loci were previously used (individual assignment, relatedness, population/landscape genetics, panel review, phylogenetics, or forensics) to determine the potential utility of these markers for common genetic analyses. Finally, we collected the following information in order to assess the efficacy of each marker: (1) evidence of null and/or dropped alleles, (2) imperfect repeats, (3) deviations from Hardy-Weinberg expectations, and (4) evidence for linkage disequilibrium. Microsatellite markers were also chosen, in part, with input from other research groups to ensure that results could be compared across studies (West Virginia University Wild Genomics Lab; Iowa State University Wildlife Health and Genetics Lab).

### Evaluation of microsatellite panel

**Fig. 1:**
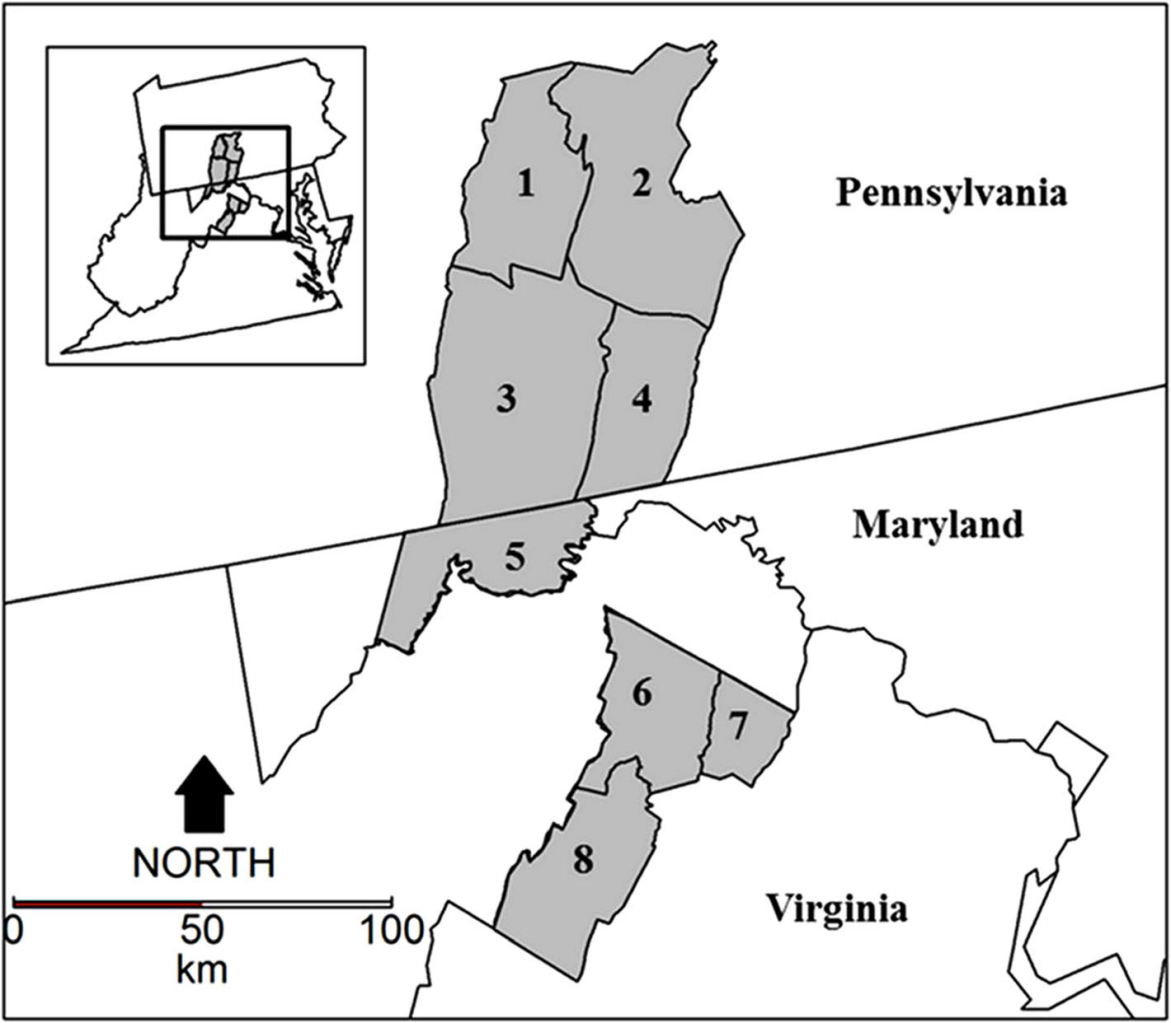
Map of white-tailed deer sampling units (gray) within the Ridge-and-Valley region of Pennsylvania, Maryland, and Virginia that were used to evaluate the described microsatellite panel. Sampling areas are numerically labeled in a north-to-south direction. The inset map shows the relative locations of the study area within the Mid-Atlantic region of the United States of America. Figure generated with ArcMap 10.2 (www.esri.com) and RStudio (version 1.1.456; www.rstudio.com). County outlines were adapted from the USGS Small-scale Dataset – 2000 County Boundaries of the United States 200,506 Shapefile (Source: U.S. Geological Survey). State boundaries were adapted from the USA States (Generalized) layer available from Esri (Source: Esri, TomTom, U.S. Department of Commerce, U.S. Census Bureau)

We collected 720 tissue samples from white-tailed deer from an area of about 25,000 km^2^, encompassing Pennsylvania, Virginia, and Maryland (Fig. 1). A total of eight counties were sampled within the region, with sample sizes ranging from 24 to 183 individuals per county. Tissue samples were selected from a single ecophysiographic province (Ridge-and-Valley region). We chose to focus on a single province to reduce the possibility of grouping together samples collected from two genetically distinct populations. Tissue samples consisted of either muscle biopsies or ear punches. These samples were collected in conjunction with routine disease surveillance efforts led by cooperating management agencies and consisted of hunter harvest and road-kill specimens. Samples were suspended in 95% ethanol and stored in a 0 °C freezer.

Genomic DNA was extracted using QIAGEN DNeasy blood and tissue extraction kits (QIAGEN, Valencia, CA, USA) following the protocol outlined for DNA isolation from animal tissues. The following modifications were made to the standard protocol: (1) tissue digestions were incubated for a minimum of four hours to ensure samples were completely lysed; (2) DNA elutions were carried out with a single 150 μL volume of elution buffer in order to maximize DNA concentration. We quantified the concentration of extracted DNA (ng/ μL) using a NanoDrop spectrophotometer (ThermoFisher Scientific, Waltham, MA, USA). Stock DNA was diluted to 20 ng/ μL prior to PCR amplification of microsatellite loci.

Polymerase chain reaction amplification of microsatellite loci was performed using fluorescently-labeled forward primers and unlabeled reverse primers (Additional file 1: Table S1). We optimized PCR conditions for use with the QIAGEN Multiplex PCR Kit (QIAGEN, Valencia, CA, USA). Microsatellite primers were multiplexed based on optimized annealing temperature, allele size distribution, dye color, and stutter pattern to ensure efficiency and minimize genotyping errors. A total of four PCR multiplexes were identified (Additional file 1: Table S1). Total reaction volumes amounted to 10.00 μL: 5.00 μL 2x QIAGEN Multiplex PCR Master Mix, 1.00 μL 5x Q-Solution, multiplex primer cocktail with a total volume equal to the sum of individual primer volumes (Additional file 1: Table S1), 1.00 μL of 20 ng/μL DNA template, and deionized H_2_O to 10 μL. Reaction conditions were adapted from the manufacturer’s recommended conditions: 95 °C for 15 min; 35x (94 °C for 30 s, multiplex-specific annealing temperature for 90 s, and 72 °C for 60 s); and a final extension of 72 °C for 10 min. Locus Cervid 1 exhibited large stutter peaks using this protocol, so it was amplified separately with 30 cycles and was then included in the multiplex for fragment size analysis.

The PCR amplicons (1 μL each) were loaded into individual wells of a 96-well sample plate and mixed with 10 μL of a denaturing agent (Hi-Di Formamide; ThermoFisher Scientific, Waltham, MA, USA). One negative control (deionized H_2_O) was included on each plate to ensure PCR amplicons were not contaminated by external sources of DNA. Three previously genotyped samples were included on each plate to validate that microsatellite calls were reproducible. Amplicons were visualized using an Applied Biosystems genetic analyzer (model 3730 XL; Waltham, MA, USA) at the Penn State Genomics Core Facility (University Park, PA, USA). As a final measure of data quality, we estimated the scoring error rate by re-extracting and reamplifying 71 randomly chosen samples (approximately 10% of the total sample size). Error rates were calculated for the overall sample and per locus as the number of miscalled alleles divided by the total number of scored alleles.

We used GeneMarker (Softgenetics, State College, PA, USA) to determine allele identity based on a known DNA size standard (GeneScan™ 500 LIZ™ Dye Size Standard; ThermoFisher Scientific, Waltham, MA, USA). GeneMarker returns a continuous fragment size value, while most tools used to analyze microsatellite data require discrete values. This introduces a potential source of error related to the subjective interpretation of microsatellite electropherograms. Given the considerable number of research studies focused on white-tailed deer genetics (Additional file 2: Table S2; Additional file 3: Appendix 1), we believe that automated binning software presents a potential tool for reducing subjectivity and improving the ability of research groups to compare results. We used program R (version 3.4.3) to automate the allele binning process [38]. The MsatAllele package was used to create cumulative fragment size distributions, histograms, and proposed bin ranges for alleles, which in turn were used to guide the determination of allele bin ranges [30]. Histograms were used to identify suspected alleles, rather than reported repeat motif, in order to account for the potential presence of imperfect repeat mutations. Allele calls were independently verified by two individuals trained in microsatellite analysis following the automated binning procedure.

Pairwise F_ST_ values were calculated between sampling units (counties) to test for the presence of possible substructure within the sampling region, which may explain deviations from Hardy-Weinberg assumptions in the pooled sample. Pairwise F_ST_ values were estimated using FSTAT (version 6.5) [39]. Significance of pairwise F_ST_ estimates was evaluated using a nominal level of 1/100 for multiple tests, which corresponded to 2800 random permutations of the data, and a Holm’s sequential Bonferroni correction [40, 41]. Null allele frequencies were estimated using the EM algorithm, implemented in FreeNA [42]. We tested for linkage disequilibrium and deviations from Hardy-Weinberg expectations using exact tests, performed in Genepop (version 2.9.3.2) [43, 44]. *P*-values were estimated using a Markov chain method and significance was assessed using Holm’s sequential Bonferroni correction [40, 41]. We used GenAlEx (version 6.5) to summarize the number of alleles per locus, observed heterozygosity (H_O_), and unbiased expected heterozygosity (H_E_) [45, 46]. Null allele frequencies, tests for linkage disequilibrium and deviations from Hardy-Weinberg proportions, and measures of genetic diversity were calculated for each sampling unit and for the pooled sample. Program CERVUS was used to determine the polymorphic information content (PIC) of each locus and the probability of identity for multi-locus genotypes [47, 48].

## Results

### Review of microsatellite studies

**Fig. 2:**
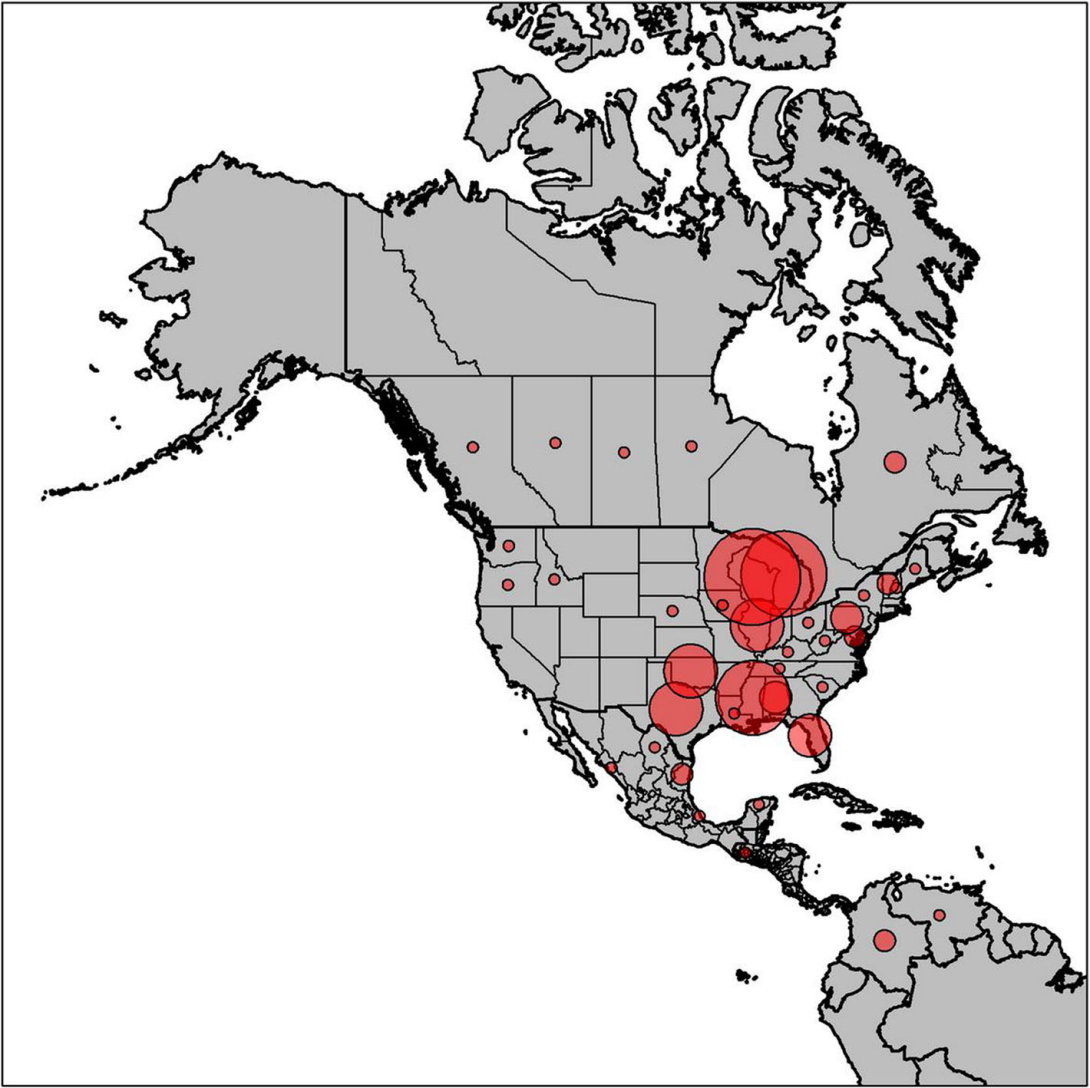
The geographic distribution of reviewed studies located within the native range of white-tailed deer. Circles overlay the state or province where previous studies occurred. Circle size corresponds to the number of genetic studies on white-tailed deer originating from those locations. Studies incorporating samples from multiple states or provinces were counted individually for each location. Additional studies from managed herds or zoological parks from Finland, Germany, and New Zealand were also reviewed but were not mapped since they occurred outside of the native range of white-tailed deer. Figure generated with ArcMap 10.2 (www.esri.com) and RStudio (version 1.1.456; www.rstudio.com). Boundaries of North America were made using Natural Earth (Free vector and raster map data available at www.naturalearthdata.com). Boundaries of South America were made using data available from Orogénesis Geographic Solutions (Source: www.tapiquen-sig.jimdo.com)

We reviewed 55 peer-reviewed papers that used microsatellite loci to study aspects of white-tailed deer genetics from 2002 to 2017 (Additional file 2: Table S2; Additional file 3: Appendix 1). We also included three additional microsatellite panels from ongoing studies in Pennsylvania, New York, and New England, as there were few published studies on white-tailed deer genetics from those regions. The geographic distribution of papers spans the range of white-tailed deer in North America, with panels included from four countries (United States of America, Canada, Mexico, and Guatemala), 24 states from the United States of America, five Canadian provinces, and five Mexican states (Fig. 2). In South America, panels were reviewed from Columbia and Venezuela. Five additional panels were reviewed from international captive and zoo populations, which included two from Finland, one from Germany, and one from New Zealand. The most common application was population genetic analyses (22) followed by the evaluation of parentage and genetic relatedness (21). Other applications included: forensic analyses (1), individual identification (3), microsatellite panel development and evaluation (8), and phylogenetics (1). Additionally, one study focused on association mapping using a large suite of bovine microsatellites [49]. Because these loci are unique and specific to this task, we elected to exclude it from consideration for further analyses.

A total of 106 microsatellite loci are reported from the reviewed papers, although some studies did test other loci that went unreported. Four loci (BM42, SRCSP1, FCB193, and OBCAM) are reported from a limited number of studies and seem to be different labels for more commonly used names (BL42, SRCSP-10, OarFCB193, and OCAM). No unique sequence was reported with these markers and they were not considered independently in the final panel. A total of 27 loci were used in ≥10 studies (Additional file 2: Table S2). Nine of these loci showed some evidence of null and/or dropped alleles (frequency > 10%) and 10 exhibited signs of imperfect repeat motifs.

### Panel selection and evaluation

We evaluated a subset of 17 microsatellite markers selected from the most commonly used microsatellites from this review (Additional file 1: Table S1). Fifteen were chosen because they were commonly used (in 10 or more previous studies) across the range of white-tailed deer (Additional file 2: Table S2). Two additional loci were chosen (RT23 and BL42) because of the positive results after being used in current studies of white-tailed deer population genetics in other laboratory groups. Successful and consistent PCR amplification was achieved for 16 out of 17 loci. RT23 exhibited amplification of non-specific products (e.g. three peaks in microsatellite profile) that was consistent, replicable, and did not respond to procedures meant to reduce non-specific binding, such as touchdown PCR, and was removed from subsequent analyses. We were able to genotype all individuals across the remaining 16 loci in 99.9% of cases.

We found a low rate of genotyping error (0.4%), defined by miscalled alleles in the original database divided by the total number of alleles, across the entire panel following comparison with 71 reamplified samples. Genotyping error rates exceeded 1.0% for loci BM848 and D (2.1% each). Only one error was attributed to user interpretation (e.g. an allele that was wrongly designated), indicating that errors related to user interpretation are likely minimal. All other instances of genotyping error corresponded to small electrophoretic peaks that were present in one run but absent in another, which indicated that these errors are more likely to result from PCR anomalies (e.g. null alleles). We did find significant evidence for null alleles, as highlighted by estimated null allele frequencies > 10% in five out of 16 microsatellite loci (Table 1). Allele frequencies at nine loci deviated significantly from Hardy-Weinberg expectations (Table 1). Two loci showed evidence of imperfect repeat motifs (loci N and Q).

**Table 1:** Genetic summary statistics averaged across eight white-tailed deer collection units

Locus	N	Range	PIC	H_O_	H_E_	NAF	HWE	LD
Multiplex 1								
RT9	13	102–125	0.8367	0.841	0.848	0.8%	0/8	BM6438
BM4107	16	134–166	0.856	0.862	0.865	0.8%	0/8	NS
P	9	210–244	0.842	0.763	0.849	4.0%	0/8^e^	NS
N	26^b^	284–380	0.922	0.691	0.913	11.1%^d^	5/8^e^	NS
Cervid1	19	159–196	0.857	0.726	0.872	6.5%^c^	2/8^e^	NS
Multiplex 2								
BM6506	16	172–213	0.906	0.674	0.903	11.8%^d^	5/8^e^	N
Q	20^b^	228–295	0.917	0.851	0.911	2.7%	0/8^e^	NS
BM848	17	362–394	0.887	0.532	0.883	18.2%^d^	7/8^e^	NS
D	12	154–198	0.786	0.534	0.804	14.6%^d^	6/8^e^	NS
Multiplex 3								
BM4208	23	140–185	0.915	0.639	0.906	13.7%^d^	7/8^e^	NS
RT7	19	207–243	0.873	0.806	0.877	4.3%	0/8^e^	NS
BM6438	15	251–280	0.884	0.841	0.885	2.6%	0/8	RT9
INRA011	8	189–207	0.492	0.522	0.522	0.5%	0/8	NS
Multiplex 4								
RT5	14	98–125	0.884	0.873	0.885	1.0%	0/8	NS
OarFCB193	18	90–127	0.885	0.871	0.878	0.6%	0/8	NS
BL42	14	235–266	0.822	0.819	0.807	0.1%	0/8	NS
RT23^a^	–	–	–	–	–	–	–	–

*N* number of alleles, *Range* allele size range, *PIC* polymorphic information criterion, *H*_*O*_ observed heterozygosity, *H*_*E*_ unbiased expected heterozygosity, *NAF* null allele frequency, *HWE* number of populations deviating from Hardy-Weinberg expectations, *LD* loci in linkage disequilibrium

^a^ Large nonspecific product present, making results uninterpretable. ^b^ Evidence of imperfect repeat motif. ^c^Null allele frequency ≥ 5% at a regional scale. ^d^Null allele frequency ≥ 10% at a regional scale. ^e^ Significant deviations from Hardy-Weinberg expectations at a regional scale

We did detect evidence of population substructure (Table 2), which may suggest that deviations from Hardy-Weinberg expectations observed in the pooled sample were caused by the population substructure. We partitioned the population by county (Fig. 1) and reevaluated Hardy-Weinberg assumptions. Significant deviations from Hardy-Weinberg proportions were observed in 25.0% of population/loci comparisons (Table 1). Five of the nine loci that deviated from expected proportions in the pooled sample accounted for 30 out of the 32 population/loci comparisons deviating from equilibrium assumptions. Continued deviations from expected proportions at these five loci were consistent with the high frequency of null alleles observed (Table 1). After these five loci were removed, we found that 97.3% of loci/population comparisons were in Hardy-Weinberg equilibrium, suggesting that deviations from expected proportions at the remaining four loci were likely caused by substructure. Genetic linkage was detected for one pair of loci (loci RT9 and BM6438; 0.2% of all comparisons including the 11 remaining loci). This relationship was significant in only one population, thus we determined that these loci are most likely independent of each other. Genetic summary statistics indicated that the 11-marker panel exhibited moderate to high levels of polymorphism (PIC = 0.492–0.917), heterozygosity (H_E_ = 0.522–0.919), and allelic richness (8–20 alleles per locus; Table 2). The probability of identity estimated from this microsatellite panel was 2.915E^− 17^. The remaining loci were consolidated into three multiplex panels with updated reaction conditions following the removal of the five problematic loci (Table 3).

**Table 2:** F_ST_ values (below diagonal) and significance tests (above diagonal) measured among white-tailed deer sampling units

	1	2	3	4	5	6	7	8
Full Panel								
1	0	*	*	*	*	*	*	*
2	0.0025	0	*	*	*	*	*	*
3	0.0050	0.0060	0	NS	*	*	*	*
4	0.0105	0.0081	0.0024	0	NS	*	*	*
5	0.0114	0.0137	0.0033	0.0015	0	*	*	*
6	0.0177	0.0211	0.0160	0.0131	0.0110	0	*	*
7	0.0114	0.0182	0.0118	0.0091	0.0095	0.0042	0	*
8	0.0269	0.0331	0.0257	0.0253	0.0207	0.0085	0.0109	0
Final Panel								
1	0	*	*	*	*	*	*	*
2	0.0021	0	*	*	*	*	*	*
3	0.0055	0.0071	0	NS	*	*	*	*
4	0.0103	0.0106	0.0031	0	NS	*	*	*
5	0.0103	0.0135	0.0035	0.0016	0	*	*	*
6	0.0204	0.0243	0.0191	0.0142	0.0136	0	NS	*
7	0.0122	0.0199	0.0136	0.0090	0.0089	0.0055	0	*
8	0.0304	0.0377	0.0293	0.0269	0.0220	0.0107	0.0116	0

All sampling units were from three states (Pennsylvania, Maryland, and Virginia) from the Mid-Atlantic region of the United States of America. Unit designations correspond to those outlined in Fig. 1. F_ST_ values that were significantly different from zero after a Holm-Bonferroni correction for multiple comparisons are designated with an asterisk (*). All non-significant tests are designated with an NS

**Table 3:** Multiplex and reaction conditions for optimized white-tailed deer microsatellite panel

Locus	Primer Sequence	Motif	Vol	Dye	AT
Multiplex 1					
RT9	F: TGAAGTTTAATTTCCACTCT R: CAGTCACTTTCATCCCACAT	2	0.20	6-FAM	57.0
BM4107	F: AGCCCCTGCTATTGTGTGAG R: ATAGGCTTTGCATTGTTCAGG	2	0.18	6-FAM	57.0
P	F: TTTCACTGTTTTCTCCTTCAGA R: TGCCCAATCAGATGTTGTAG	4	0.20	NED	57.0
Cervid 1^†^	F: AAATGACAACCCGCTCCAGTATC R: TCCGTGCATCTCAACATGAGTTAG	2	0.15	NED	64.0
Multiplex 2					
Q	F: AATGTGTCAGTGAAGGTCTTC R: ATCCAGGCAACCATCTAG	4	0.18	6-FAM	60.0
RT5	F: CAGCATAATTCTGACAAGTG R: GTTGAGGGGACTCGACTG	2	0.16	6-FAM	60.0
BL42^†^	F: ACAAGTCAAGGTCAAGTCCAAATGCC R: CGATTTTTGTGTTAATTTCATGC	2	0.20	PET	54.0
Multiplex 3					
RT7	F: CCTGTTCTACTCTTCTTCTC R: ACTTTTCACGGGCACTGGTT	2	0.15	VIC	55.0
BM6438	F: TTGAGCACAGACACAGACTGG R: ACTGAATGCCTCCTTTGTGC	2	0.17	NED	55.0
INRA011	F: CGAGTTTCTTTCCTCGTGGTAGGC R: GCTCGGCACATCTTCCTTAGCAAC	2	0.17	PET	55.0
OarFCB193	F: TTCATCTCAGACTGGGATTCAGA R: GCTTGGAAATAACCCTCCTGC	2	0.18	NED	55.0

All primers were multiplexed for PCR and fragment analysis steps with the exception of Cervid 1 and BL42 (^†^). Polymerase chain reactions for these two loci were run separately and then multiplexed for fragment size analysis. Primers are grouped by multiplex with Motif = expected repeat motifs, Vol = volume of primer mix (μL) for 10 μL reaction volume consisting of equal parts 20 μM forward and reverse primer, Dye = dye color, and AT = annealing temperature (°C)

## Discussion

### Panel selection and evaluation

Our literature review produced a list of 106 microsatellite loci, a number that is excessive and impractical for most study objectives. A smaller proportion of these loci, however, were used in previous studies across a significant proportion of the North American range of white-tailed deer and represent appropriate candidates for a core microsatellite panel. We chose to test 17 loci, 15 of which were widely used among the studies reviewed. Two additional loci were tested because other laboratories are currently using these markers and we wanted to maximize our ability to compare results across studies.

We found significant deviations from Hardy-Weinberg expectations at nine loci in the full population (loci P, N, Cervid 1, BM6506, BM848, Q, D, BM4208, and RT7). Null and/or dropped allele frequencies exceeded 10% for five of these markers (loci N, BM6506, BM848, D, and BM4208) indicating that deviations from Hardy-Weinberg equilibrium were most likely related to genotyping errors. Statistical corrections (re-estimation of allele frequencies after accounting for null and/or alleles) can improve bias caused by amplification errors, although this is not an absolute solution for these sources of bias [28]. Because null allele frequencies exceeded 10% for these five loci, we elected to remove these loci from the final panel rather than use available software to provide ‘corrected’ genotypes. We found evidence of population substructure that may have accounted for deviations from Hardy-Weinberg equilibrium in the remaining four loci (P, Cervid 1, Q, and RT7). After stratifying our region by county, only a small subset of subpopulations deviated from Hardy-Weinberg equilibrium for one of these loci (Cervid 1; two out of eight populations). These results are in concordance with deviations observed in DeYoung et al. [36], which suggests that population substructure is the most likely reason for deviations at this locus.

**Fig. 3:**
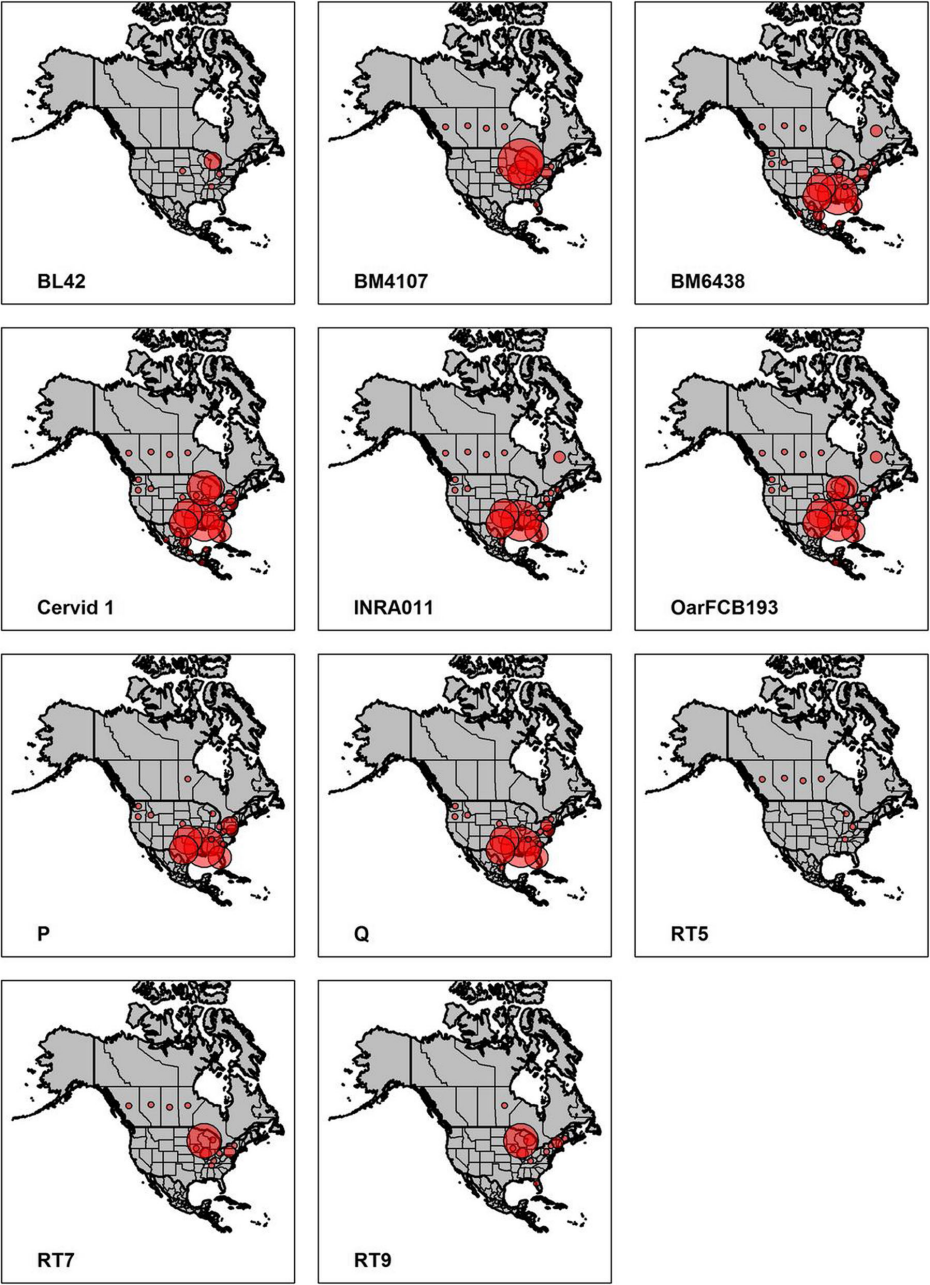
The geographic distribution of reviewed studies that used loci included in the final 11 microsatellite panel. Circles overlay the state or province where previous studies occurred. Circle size corresponds to the number of genetic studies on white-tailed deer originating from those locations. Studies incorporating samples from multiple states or provinces were counted individually for each location. Figure generated with ArcMap 10.2 (www.esri.com) and RStudio (version 1.1.456; www.rstudio.com). Boundaries of North America were made using Natural Earth (Free vector and raster map data available at www.naturalearthdata.com)

The remaining 11 loci were found to amplify consistently, exhibit high rates of polymorphism, have low rates of inferred genotyping errors, and were able to be multiplexed effectively. Evidence for genetic linkage between loci was minimal in this and previous studies, indicating that deviations from linkage equilibrium are rare. It is likely that these markers represent genetically-independent units in most situations. Additionally, nine of these loci have been used in many studies conducted across the broad geographic range of white-tailed deer indicating their potential for widespread application in comparative and collaborative studies (Fig. 3). For the most part, all loci were easily interpretable, although, one locus did exhibit evidence of imperfect repeats (e.g. locus Q) consistent with allele frequency distributions from previous studies [28, 36]. Fortunately, incomplete repeats in this panel arose in tetranucleotide loci, making their interpretation easier. Several other loci were highly polymorphic, which led to deviations from the expected allele repeat motif due, in large part, to differences in electrophoretic motility (e.g. alleles were 1.8 base pairs apart compared to two). Failing to account for imperfect repeats and differences in motility can lead to call mismatches and deflated estimates of heterozygosity, potentially biasing genetic results [29]. While allele binning software is a common method of identifying allele identity, many available programs bin fragment sizes to allele designations based on repeat motif. Programs that allow for irregular calls, such as MsatAllele [30], may assist in reducing errors related to imperfect repeat mutations and their adoption into standard methodologies is likely to benefit future studies. Although time consuming, re-checking all loci manually following allele binning may further ensure the accuracy of allele calls. Additionally, two loci (Cervid 1 and P) also have alleles that do not amplify strongly in heterozygous individuals. We chose to score electropherogram peaks that exhibited < 50% amplitude compared to the second allele peak if small peaks were replicable and showed similar stutter patterns. Inclusion of these peaks produced results that better matched Hardy-Weinberg expectations as compared to exclusion. Other studies also report modified versions of the locus P primer that increased primer-binding efficiency [50].

The microsatellite panel described here likely would largely be applicable across research objectives that utilize high-quality genetic samples. All loci included in the final panel exhibit null allele frequencies < 10%, which is acceptable for population genetics analyses [28]. While not excessive, locus Cervid 1 was characterized by an average null allele frequency of 6.5% (Table 1). While this marker is not expected to bias population genetics analyses, it may produce more common homozygous mismatches between parents and offspring compared to the other loci in the panel with null allele frequencies under 5% [27, 28]. Many statistical packages used to evaluate parentage, such as CERVUS, provide methods that correct for the infrequent occurrence of null alleles when calculating exclusion probabilities [47, 48]. Therefore, null alleles occurring at this frequency in a single locus are not expected to influence most genetic analyses when appropriate measures are used to account for their presence.

### Limitations and future considerations

One objective of this study was to formalize a protocol for marker selection based on the geographic range of previous studies that can be adopted for this species and other commonly studied species. While this procedure addresses several methodological limitations outlined by Moran et al. [26], one drawback is that this methodology relies on previous studies to self-report genotyping errors. We found that only about 7% of studies reported high frequencies of common genotyping errors in loci where we detected null and/or dropped alleles, which seemingly indicated that genotyping errors were rare. This low rate of genotyping error, however, may also be attributed to the fact that genotyping errors went underreported. Only 22% of studies reported explicitly testing for the presence of amplification errors. Most studies inferred the presence of amplification errors from deviations from Hardy-Weinberg expectations instead. But, as observed here and by others [36], deviations from Hardy-Weinberg assumptions may also be caused by population substructure in white-tailed deer populations. Therefore, it would be desirable for future studies to provide an explicit evaluation of potential sources of genotyping errors (e.g. statistical tests, such as FreeNA [42], evaluation of known dam – sire relationships, or locus sequencing) that may bias results or lead to the exclusion of specific markers. Additionally, many studies fail to report the details of their marker selection process, often indicating that they tested unnamed loci that were otherwise unmentioned in the paper. While this information may not be relevant to the conclusions made in any one study, explicitly detailing the full number of microsatellites tested and the reaction conditions used is of great benefit during the marker selection stage of future studies.

Only high-quality DNA sources (e.g. connective tissue biopsies) were used in this and most previous microsatellite panel evaluations. The use of low-quality DNA sources, such as hair and scat, is becoming increasingly prevalent and is of interest to future studies. These sources of DNA often have higher rates of dropped alleles due to degradation of the DNA template or the presence of PCR inhibitors [51, 52]. Thus, microsatellites that are optimized for use with high-quality DNA sources may not be generalizable to low-quality sample types. Our literature review indicated that all loci, except for locus RT9, have been used in previous studies incorporating low-quality sample types [53–55]. Only locus BL42 was found to have very minor genotyping errors [54]. Thus, this panel is expected to be useful for situation where non-invasive genetic sampling is necessary (e.g. genetic-based mark-recapture studies) or in cases where high-quality DNA may not be available (e.g. forensic analyses). An effort to assess the effective use of this panel for low-quality DNA sources is an area of ongoing research.

Despite the importance of identifying a core microsatellite panel for comparative use, one challenge that remains to developing standardized analytical pipelines is the difficulty in reproducing allele calls. This is an inherent methodological challenge of genotyping efforts based on microsatellite loci and is most commonly caused by differences in genotyping platforms, PCR conditions, and electrophoretic mobility of reagents [22, 26, 32, 33]. These differences can impede the ability of researchers to pool data. However, these differences do not limit the ability to compare results, such as patterns of genetic diversity and population structure. Shifts in fragment size distributions related to methodological differences should not affect the occurrence or identity of alleles. Thus, summary measures, such as heterozygosity and allelic richness, should still be comparable among laboratory groups even if allele designations differ if a consistent methodology for identifying and calling unique alleles is adopted. This highlights the importance of developing core microsatellite panels with straightforward methodologies, such as the one described here. The development and adoption of standardization practices that would allow for the pooling of data, such as the use of internal size standards to align fragment size distributions [22], is a logical extension this research, but an effort that would be more difficult without first identifying a core panel and methodology.

Currently, there is increased interest in producing SNP panels for commonly studied species, which has been facilitated by the proliferation of next-generation sequencing technologies. Despite the increased focus on SNPs, it is likely that microsatellites will remain an important tool for studying the genetics of many species. While SNP genotyping can account for concerns regarding data reproducibility and intergroup comparisons, they are more expensive and require more advanced bioinformatical pipelines to process and analyze data. In many cases, SNPs provide only marginal benefits over microsatellites for many common research objectives despite the increase in cost. For example, a previous assay containing 878 polymorphic SNP loci for white-tailed deer and mule deer (*O. hemionus*) had similar power to discern population structure and phylogenetic relationships when compared to 10 highly polymorphic microsatellite loci [56]. Given the higher cost and marginal gains in power of alternative marker types, it is likely that microsatellites will remain an important tool for studying genetic aspects of species of management interest, such as white-tailed deer. Focus on the development of uniform panels, methodologies, and best management practices for microsatellite markers is likely to benefit efforts to improve reproducibility and the ability to compare results among research groups, areas where SNP panels currently have some benefit over microsatellites.

## Conclusions

Given that microsatellites are expected to be a useful tool for the study of non-model species, identification of core marker panels and standard methodologies can benefit genetic studies of common and widely-distributed species. We have identified a microsatellite panel for genetic analysis of white-tailed deer that is likely to be broadly applicable across this species’ range based on the geographic distribution of previous studies that used these loci (Fig. 3). Assessment of the effectiveness of these markers in a population from the Mid-Atlantic region of the United States of America, an area with few previous studies, provided further evidence for the conservation of these sequences and their utility in novel populations. The identification of core microsatellite panels and detailed methodological pipelines represents an important step forward in improving the repeatability and comparison of genetic results among research groups. While many laboratory groups choose markers from previous studies in order to maintain continuity between studies, it is still common for laboratory groups to utilize divergent panels of loci, even within the same region. For example, two overlapping studies of deer from the lower peninsula of Michigan, USA, utilized different suites of loci even though they were focusing on studying genetic characteristics of the same population [57, 58]. Out of 11 total loci used in these studies, only three were shared among the two panels. Formal reevaluations of commonly used genetic resources and adoption of a core microsatellite panel would improve the ability to compare results between future studies in this species.

While our literature review suggests that these markers are characterized by low rates of genotyping errors across many regions of this species’ range, this study is not meant to replace a careful assessment of panel efficacy. In fact, genotyping errors, such as null or partially amplifying alleles, may be underreported in previous literature. Further, microsatellite loci may still exhibit population-specific idiosyncrasies, and testing for the presence of genotyping errors at the onset of new studies is still warranted. Rather, the impetus for this panel was to identify a core set of markers and develop a standard methodology that can act as a starting point for future studies of this species and to provide a uniform platform to support collaborative efforts and comparisons. While some specific applications, like linkage mapping, may require the use of additional loci, we feel that this core panel is adequate for many of the most common genetic analyses and provides a base for additional applications, such as analyses of low-quality DNA samples. The identification of core panels further benefits the development of standardization practices, which can further improve repeatability and collaborative efforts and are a logical extension of this and similar studies.

## Additional files

Additional file 1: **Table S1.** Multiplex and reaction conditions for all white-tailed deer microsatellite loci evaluated. (DOCX 19 kb)

Additional file 2: **Table S2.** Summary of microsatellite loci used in previous studies of white-tailed deer genetics. Studies are arranged by date. Study area(s) are also indicated and grouped by state (if in North America) and Country. Loci included in the final microsatellite panel are highlighted in blue (XLSX 35 kb)

Additional file 3: **Appendix 1.** Citations corresponding to all reviewed studies listed in Additional file 2: Table S2. (DOCX 19 kb)

## Data Availability

The microsatellite genotypes generated and analyzed during the current study are not publicly available due to ongoing work on the genetics of these populations but are available from the corresponding author on reasonable request. R script for binning raw allele scores is distributed under the GPL-3 license and is available at http://ecosystems.psu.edu/research/labs/walter-lab/additional-labs/population-genetics-lab. Script was prepared using R versions 3.4.3 - 3.5.0 on the Microsoft Windows platform (Windows 7 Enterprise edition).

## Abbreviations

AFLP: Amplified fragment length polymorphism
H_E_: Expected heterozygosity
H_O_: Observed heterozygosity
PCR: Polymerase chain reaction
PIC: Polymorphic information content
SNP: Single nucleotide polymorphism
