# Supplementary material for: Identification and evaluation of a core microsatellite panel for use in white-tailed deer (Odocoileus virginianus)
Source: BMC Genet. 2019 Jun 6;20:49. doi: 10.1186/s12863-019-0750-z (PMC6554959; doi:10.1186/s12863-019-0750-z)
Supplement: Supplementary file 1 — Table S1. Multiplex and reaction conditions for all white-tailed deer microsatellite loci evaluated. (DOCX 19 kb) [file 12863_2019_750_MOESM1_ESM.docx]

**Table S1** Multiplex and reaction conditions for all white-tailed deer microsatellite loci evaluated

| **Locus** | **Primer Sequence** | **Motif** | **Vol** | **Dye** | **AT** | | **Ref** | |
| --- | --- | --- | --- | --- | --- | --- | --- | --- |
| **Multiplex 1** | | | | | |  | |  |
| RT9 | F: TGAAGTTTAATTTCCACTCT  R: CAGTCACTTTCATCCCACAT | 2 | 0.20 | 6-FAM | 57.0 | | [1] | |
| BM4107 | F: AGCCCCTGCTATTGTGTGAG  R: ATAGGCTTTGCATTGTTCAGG | 2 | 0.18 | 6-FAM | 57.0 | | [2] | |
| P | F: TTTCACTGTTTTCTCCTTCAGA  R: TGCCCAATCAGATGTTGTAG | 4 | 0.20 | NED | 57.0 | | [3] | |
| N | F: TCCAGAGAAGCAACCAATAG  R: GTGTGCCTTAAACAACCTGT | 4 | 0.16 | PET | 57.0 | | [3] | |
| Cervid 1^†^ | F: AAATGACAACCCGCTCCAGTATC  R: TCCGTGCATCTCAACATGAGTTAG | 2 | 0.15 | NED | 64.0 | | [4] | |
| **Multiplex 2** | | | | | |  | |  |
| BM6506 | F: GCACGTGGTAAAGAGATGGC  R: AGCAACTTGAGCATGGCAC | 2 | 0.17 | 6-FAM | 60.0 | | [2] | |
| BM848 | F: TGGTTGGAAGGAAAACTTGG  R: CCTCTGCTCCTCAAGACAC | 2 | 0.18 | 6-FAM | 60.0 | | [2] | |
| Q | F: AATGTGTCAGTGAAGGTCTTC  R: ATCCAGGCAACCATCTAG | 4 | 0.18 | 6-FAM | 60.0 | | [3] | |
| D | F: AGAGCCTCGTCTTTTCATTC  R: TTGCTGCTTGCTTGTCTAAT | 4 | 0.17 | PET | 60.0 | | [3] | |
| **Multiplex 3** | | | | | |  | |  |
| RT7 | F: CCTGTTCTACTCTTCTTCTC  R: ACTTTTCACGGGCACTGGTT | 2 | 0.16 | VIC | 55.5 | | [1] | |
| BM6438 | F: TTGAGCACAGACACAGACTGG  R: ACTGAATGCCTCCTTTGTGC | 2 | 0.20 | NED | 55.5 | | [2] | |
| BM4208 | F: TCAGTACACTGGCCACCATG  R: CACTGCATGCTTTTCCAAAC | 2 | 0.17 | VIC | 55.5 | | [2] | |
| INRA011 | F: CGAGTTTCTTTCCTCGTGGTAGGC  R: GCTCGGCACATCTTCCTTAGCAAC | 2 | 0.17 | PET | 55.5 | | [5] | |
| **Multiplex 4** | | | | | |  | |  |
| RT5 | F: CAGCATAATTCTGACAAGTG  R: GTTGAGGGGACTCGACTG | 2 | 0.16 | 6-FAM | 55.0 | | [1,7] | |
| RT23 | F: GGCCATTGGGTAGTCTCC  R: AGCCTCCCTGAGTGCTCT | 2 | 0.18 | VIC | 55.0 | | [1] | |
| OarFCB193 | F: TTCATCTCAGACTGGGATTCAGA  R: GCTTGGAAATAACCCTCCTGC | 2 | 0.18 | NED | 55.0 | | [6] | |
| BL42 | F: ACAAGTCAAGGTCAAGTCCAAATGCC  R: CGATTTTTGTGTTAATTTCATGC | 2 | 0.20 | PET | 55.0 | | [2,7] | |

All primers were multiplexed for PCR and fragment analysis steps with the exception of Cervid 1(^†^). Polymerase chain reactions for this locus were run separately and then multiplexed for fragment size analysis. Primers are grouped by multiplex with Motif = expected repeat motifs, Vol = volume of primer mix (µL) for 10 µL reaction volume consisting of equal parts 20 µM forward and reverse primer, Dye = dye color, AT = annealing temperature (˚C), and Ref = reference for primers. Primer sequences for loci RT5 and BL42 were modified from original reference as described in Lopez (2006) [7].

**References**

1. Wilson GA, Strobeck C, Wu L, Coffin JW. Characterization of microsatellite loci in caribou *Rangifer tarandus*, and their use in other artiodactyls. Mol Ecol. 1997;6:697–9.

2. Bishop MD, Kappes SM, Keele JW, Stone RT, Sunden S, Hawkins GA, et al. A genetic linkage map for cattle. Genetics. 1994;136:619–39.

3. Jones KC, Levine KF, Banks JD. DNA-based genetic markers in black-tailed and mule deer for forensic applications. Calif Fish Game. 2000;86:115–26.

4. DeWoody JA, Honeycutt RL, Skow LC. Microsatellite markers in white-tailed deer. J Hered. 1995;86:317–9.

5. Vaiman D, Osta R, Mercier D, Grohs C, Levéziel H. Characterization of five new bovine dinucleotide repeats. Anim Genet. 1992;23:537–41.

6. Buchanan FC, Crawford AM. Ovine microsatellites at the OarFCB11, OarFCB128, OarFCB193, OarFCB266 and OarFCB304 loci. Anim Genet. 1993;24:145.

7. Lopez RG. Genetic structuring of Coues white-tailed deer in the southwestern United States [master’s thesis]. Flagstaff, AZ: Arizona State University; 2006.
