# Supplementary material for: Identification and evaluation of a core microsatellite panel for use in white-tailed deer (Odocoileus virginianus)
Source: BMC Genet. 2019 Jun 6;20:49. doi: 10.1186/s12863-019-0750-z (PMC6554959; doi:10.1186/s12863-019-0750-z)
Supplement: Supplementary file 3 — Appendix 1. Citations corresponding to all reviewed studies listed in Additional file 2: Table S2. (DOCX 19 kb) [file 12863_2019_750_MOESM3_ESM.docx]

**Appendix 1** Citations corresponding to all reviewed studies listed in Table S2

**LITERATURE CITED**

1. Anderson JD, Honeycutt RL, Gonzales RA, Gee KL, Skow LC, Gallagher RL, et al. Development of microsatellite DNA markers for the automated genetic characterization of white-tailed deer populations. J Wildl Manage. 2002;66:67–74.

2. DeYoung RW, Demarais S, Gonzales RA, Honeycutt RL, Gee KL. Multiple paternity in white-tailed deer (*Odocoileus virginianus*) revealed by DNA microsatellites. J Mammal. 2002;83:884–92.

3. Smith PF, DenDanto D, Smith KT, Palman D, Kornfield I. Allele frequencies for three STR loci RT24, RT09, and BM1225 in northern New England white-tailed deer. J Forensic Sci. 2002;47:673–675.

4. Blanchong JA, Scribner KT, Winterstein SR. Assignment of individuals to populations: Bayesian methods and multi-locus genotypes. J Wildl Manage. 2002;66:321–9.

5. DeYoung RW, Demarais S, Honeycutt RL, Gonzales RA, Gee KL, Anderson JD. Evaluation of a DNA microsatellite panel useful for genetic exclusion studies in white-tailed deer. Wildl Soc Bull. 2003;31:220–32.

6. DeYoung RW, Demarais S, Honeycutt RL, Rooney AP, Gonzales RA, Gee KL. Genetic consequences of white-tailed deer (*Odocoileus virginianus*) restoration in Mississippi. Mol Ecol. 2003;12:3237–52.

7. Sorin AB. Paternity assignment for white-tailed deer (*Odocoileus virginianus*): mating across age classes and multiple paternity. J Mammal. 2004;85:356–62.

8. DeYoung RW, Muller LI, Demarais S, Guthrie HD, Welch GR, Engelken TJ, et al. Do *Odocoileus virginianus* males produce Y-chromosome-biased ejaculates? Implications for adaptive sex ratio theories. J Mammal. 2004;85:768–773.

9. Comer CE, Kilgo JC, D’angelo GJ, Glenn TC, Miller KV, DeWoody. Fine-scale genetic structure and social organization in female white-tailed deer. J Wildl Manage. 2005;69:332–44.

10. Doerner KC, Braden W, Cork J, Cunningham T, Rice A, Furman BJ, et al. Population genetics of resurgence: white-tailed deer in Kentucky. J Wildl Manage. 2005;69:345–55.

11. DeYoung RW, Demarais S, Honeycutt RL, Gee KL, Gonzales RA. Social dominance and male breeding success in captive white-tailed deer. Wildl Soc Bull. 2006;34:131–6.

12. Shaw JC, Lancia RA, Conner MC, Rosenberry CS. Effect of population demographics and social pressures on white-tailed deer dispersal ecology. J Wildl Manage. 2006;70:1293–301.

13. Blanchong JA, Scribner KT, Epperson BK, Winterstein SR. Changes in artificial feeding regulations impact white-tailed deer fine-scale spatial genetic structure. J Wildl Manage. 2006;70:1037–43.

14. Blanchong JA, Scribner KT, Kravchenko AN, Winterstein SR. TB-infected deer are more closely related than non-infected deer. Biol Lett. 2007;3:104–6.

15. Belant JL, Seamans TW, Paetkau D. Genetic tagging free-ranging white-tailed deer using hair snares. Ohio J Sci. 2007;107:50–6.

16. Therrien J-F, Côté SD, Festa-Bianchet M, Ouellet J-P. Conservative maternal care in an iteroparous mammal: a resource allocation experiment. Behav Ecol Sociobiol. 2007;62:193–9.

17. Ruiz-García M, Randi E, Martínez-Agüero M, Alvarez D. Phylogenetic relationships between neotropical deer genera (Ateriodactyla: Cervidae) by mitochondrial DNA sequencing and microsatellite markers. Rev Biol Trop. 2007;55:723–741.

18. Blanchong JA, Samuel MD, Scribner KT, Weckworth BV, Langenberg JA, Filcek KB. Landscape genetics and the spatial distribution of chronic wasting disease. Biol Lett. 2008;4:130–3.

19. Therrien J-F, Côté SD, Festa-Bianchet M, Ouellet J-P. Maternal care in white-tailed deer: trade-off between maintenance and reproduction under food restriction. Animal Behav. 2008;75:235–43.

20. DeYoung RW, Demarais S, Gee KL, Honeycutt RL, Hellickson MW, Gonzales RA. Molecular evaluation of the white-tailed deer (*Odocoileus virginianus*) mating system. J Mammal. 2009;90:946–953.

21. Ruiz-García M, Martinez-Agüero M, Álvarez D, Goodman S. Genetic variability in neotropical deer genera (Mammalia: Cervidae) according to microsatellite loci. Rev Biol Trop. 2009;57:879–904.

22. Blanchong JA, Heisey DM, Scribner KT, Libants SV, Johnson C, Aiken JM, et al. Genetic susceptibility to chronic wasting disease in free-ranging white-tailed deer: complement component C1q and Prnp polymorphisms. Infect Genet Evol. 2009;9:1329–35.

23. Passler T, Walz PH, Ditchkoff SS, Brock KV, DeYoung RW, Foley AM, et al. Cohabitation of pregnant white-tailed deer and cattle persistently infected with Bovine viral diarrhea virus results in persistently infected fawns. Vet Microbiol. 2009;134:362–7.

24. Rosenberry CS, Long ES, Hassel-Finnegan HM, Buonaccorsi VP, Diefenbach DR, Wallingford BD. Lack of mother–offspring relationships in white-tailed deer capture groups. J Wildl Manage. 2009;73:357–61.

25. Ernest HB, Hoar BR, Well JA, O’Rourke KI. Molecular genealogy tools for white-tailed deer with chronic wasting disease. Can J Vet Res. 2010;74:153–6.

26. Grear DA, Samuel MD, Scribner KT, Weckworth BV, Langenberg JA. Influence of genetic relatedness and spatial proximity on chronic wasting disease infection among female white-tailed deer. J Appl Ecol. 2010;47:532–40.

27. Kelly AC, Mateus-Pinilla NE, Douglas M, Douglas M, Brown W, Ruiz MO, et al. Utilizing disease surveillance to examine gene flow and dispersal in white-tailed deer. J Appl Ecol. 2010;47:1189–98.

28. Miller BF, DeYoung RW, Campbell TA, Laseter BR, Ford WM, Miller KV. Fine-scale genetic and social structuring in a central Appalachian white-tailed deer herd. J Mammal. 2010;91:681–9.

29. Passler T, Ditchkoff SS, Givens MD, Brock KV, DeYoung RW, Walz PH. Transmission of bovine viral diarrhea virus among white-tailed deer (*Odocoileus virginianus*). Vet Res. 2010;41:1, 20–8.

30. Cullingham CI, Merrill EH, Pybus MJ, Bollinger TK, Wilson GA, Coltman DW. Broad and fine-scale genetic analysis of white-tailed deer populations: estimating the relative risk of chronic wasting disease spread. Evol Appl. 2011;4:116–31.

31. Kelly AC, Mateus-Pinilla NE, Douglas M, Douglas M, Shelton P, Novakofski J. Microsatellites behaving badly: empirical evaluation of genotyping errors and subsequent impacts on population studies. Genet Mol Res. 2011;10:2534–53.

32. Keller SP, Brooks P, Huffman JE. Evaluation of the genetic variability of ten microsatellite loci in white-tailed deer (*Odocoileus virginianus*) from Monroe County, Pennsylvania. J PA Acad Sci. 2011;85:18–22.

33. Robinson SJ, Samuel MD, Lopez DL, Shelton P. The walk is never random: subtle landscape effects shape gene flow in a continuous white-tailed deer population in the Midwestern United States. Mol Ecol. 2012;21:4190–205.

34. de la Rosa-Reyna XF, Calderón-Lobato RD, Parra-Bracamonte GM, Sifuentes-Rincón AM, DeYoung RW, et al. Genetic diversity and structure among subspecies of white-tailed deer in Mexico. J Mammal. 2012;93:1158–68.

35. Blanchong JA, Grear DA, Weckworth BV, Keane DP, Scribner KT, Samuel MD. Effects of chronic wasting disease on reproduction and fawn harvest vulnerability in Wisconsin white-tailed deer. J Wildl Dis. 2012;48:361–70.

36. Kekkonen J, Wikström M, Brommer JE. Heterozygosity in an isolated population of a large mammal founded by four individuals is predicted by an individual-based genetic model. PLoS ONE. 2012;7:e43482.

37. Lang KR, Blanchong JA. Population genetic structure of white-tailed deer: understanding risk of chronic wasting disease spread. J Wildl Manage. 2012;76:832–40.

38. Blanchong JA, Sorin AB, Scribner KT. Genetic diversity and population structure in urban white-tailed deer. J Wildl Manage. 2013;77:855–62.

39. Vander Wal E, Edye I, Paquet PC, Coltman DW, Bayne E, Brook RK, et al. Juxtaposition between host population structures: implications for disease transmission in a sympatric cervid community. Evol Appl. 2013;6:1001–11.

40. Magle SB, Samuel MD, Deelen TRV, Robinson SJ, Mathews NE. Evaluating spatial overlap and relatedness of white-tailed deer in a chronic wasting disease management zone. PLoS ONE. 2013;8:e56568.

41. Goode MJ, Beaver JT, Muller LI, Clark JD, van Manen FT, Harper CA, et al. Capture—recapture of white-tailed deer using DNA from fecal pellet groups. Wildlife Biol. 2014;20:270–8.

42. Green ML, Manjerovic MB, Mateus-Pinilla N, Novakofski J. Genetic assignment tests reveal dispersal of white-tailed deer: implications for chronic wasting disease. J Mammal. 2014;95:646–54.

43. Hernández-Mendoza PM, Parra-Bracamonte GM, de la Rosa-Reyna XF, Chassin-Noria O, Sifuentes-Rincón AM. Genetic shifts in the transition from wild to farmed white-tailed deer (*Odocoileus virginianus*) population. Intl J Biodiversity Sci Eco Serv Manage. 2014;10:3–8.

44. Kelly AC, Mateus-Pinilla NE, Brown W, Ruiz MO, Douglas MR, Douglas ME, et al. Genetic assessment of environmental features that influence deer dispersal: implications for prion-infected populations. Popul Ecol. 2014;56:327–40.

45. Brommer JE, Kekkonen J, Wikström M. Using heterozygosity–fitness correlations to study inbreeding depression in an isolated population of white-tailed deer founded by few individuals. Ecol Evol. 2015;5:357–67.

46. Hopken MW, Lum TM, Meyers PM, Piaggio AJ. Molecular assessment of translocation and management of an endangered subspecies of white-tailed deer (*Odocoileus virginianus*). Conserv Genet. 2015;16:635–47.

47. Locher A, Scribner KT, Moore JA, Murphy B, Kanefsky J. Influence of landscape features on spatial genetic structure of white-tailed deer in human-altered landscapes. J Wildl Manage. 2015;79:180–94.

48. Hoffmann GS, Johannesen J, Griebeler EM. Species cross-amplification, identification and genetic variation of 17 species of deer (Cervidae) with microsatellite and mitochondrial DNA from antlers. Mol Biol Rep. 2015;42:1059–67.

49. Sumners JA, Demarais S, DeYoung RW, Honeycutt RL, Rooney AP, Gonzales RA, et al. Variable breeding dates among populations of white-tailed deer in the southern United States: the legacy of restocking? J Wildl Manage. 2015;79:1213–25.

50. Ramón-Laca A, Soriano L, Gleeson D, Godoy JA. A simple and effective method for obtaining mammal DNA from faeces. Wildlife Biol. 2015;21:195–203.

51. Neuman TJ, Newbolt CH, Ditchkoff SS, Steury TD. Microsatellites reveal plasticity in reproductive success of white-tailed deer. J Mammal. 2016;97:1441–50.

52. Turner MM, Deperno CS, Booth W, Vargo EL, Conner MC, Lancia RA. The mating system of white-tailed deer under Quality Deer Management. J Wildl Manage. 2016;80:935–40.

53. Villanova VL, Hughes PT, Hoffman EA. Combining genetic structure and demographic analyses to estimate persistence in endangered Key deer (*Odocoileus virginianus clavium*). Conserv Genet. 2017;18:1061–76.

54. Delgado ML, Singh P, Funk JA, Moore JA, Cannell EM, Kanesfsky J, et al. Intestinal microbial community dynamics of white-tailed deer (*Odocoileus virginianus*) in an agroecosystem. Microb Ecol. 2017;74:496–506.
